# Supplementary material for: Mapping the evolution of fertility support policies in China: A content and instrumental analysis
Source: PLoS One. 2025 Oct 9;20(10):e0332137. doi: 10.1371/journal.pone.0332137 (PMC12510515; doi:10.1371/journal.pone.0332137)
Supplement: S1 Appendix — (ZIP) [file pone.0332137.s001.zip › S1 Appendix. 226 original policy documents/54-国务院办公厅关于进一步释放消费潜力促进消费持续恢复的意见(FBM-CLI-2-5116175).doc]

国务院办公厅关于进一步释放消费潜力促进消费持续恢复的意见

**发布部门：** [国务院办公厅](https://www.pkulaw.com/law/chl?Aggs.IssueDepartment=601) [机构沿革](javascript:void(0))

**发文字号：**国办发〔2022〕9号

**发布日期：**2022.04.20

**实施日期：**2022.04.20

**时效性：** [现行有效](https://www.pkulaw.com/law/chl?Aggs.TimelinessDic=01)

**效力级别：** [国务院规范性文件](https://www.pkulaw.com/law/chl?Aggs.EffectivenessDic=XC0203)

**法规类别：** [消费者权益保护](https://www.pkulaw.com/law/chl?Aggs.Category=08209) [市场管理](https://www.pkulaw.com/law/chl?Aggs.Category=08204)

国务院办公厅关于进一步释放消费潜力促进消费持续恢复的意见
（国办发〔2022〕9号）

各省、自治区、直辖市人民政府，国务院各部委、各直属机构：
　　消费是最终需求，是畅通国内大循环的关键环节和重要引擎，对经济具有持久拉动力，事关保障和改善民生。当前，受新冠肺炎疫情等因素影响，消费特别是接触型消费恢复较慢，中小微企业、个体工商户和服务业领域面临较多困难。为深入贯彻习近平新时代中国特色社会主义思想，完整、准确、全面贯彻新发展理念，加快构建新发展格局，协同发力、远近兼顾，综合施策释放消费潜力，促进消费持续恢复，经国务院同意，现提出以下意见。
　　一、应对疫情影响，促进消费有序恢复发展
　　**（一）**围绕保市场主体加大助企纾困力度。深入落实扶持制造业、小微企业和个体工商户的减税退税降费政策。推动金融系统通过降低利率、减少收费等多种措施，向实体经济让利。引导金融机构优化信贷管理，对受疫情影响严重的行业企业给予融资支持，避免出现行业性限贷、抽贷、断贷。延续执行阶段性降低失业保险、工伤保险费率政策。对不裁员少裁员的企业，实施好失业保险稳岗返还政策。清理转供电环节不合理加价。采取切实有效措施制止乱收费、乱摊派、乱罚款行为。鼓励有条件的地区对零售、餐饮等行业企业免费开展员工定期核酸检测，对企业防疫、消杀支出给予补贴支持。落实好餐饮、零售、旅游、民航、公路水路铁路运输等特困行业纾困扶持措施。鼓励地方加大帮扶力度，支持各地区结合实际依法出台税费减免等措施，对特困行业实行用电阶段性优惠、暂缓缴纳养老保险费等政策，对承租非国有房屋的服务业小微企业和个体工商户给予适当帮扶，稳住更多消费服务市场主体。

　　**（二）**做好基本消费品保供稳价。结合疫情防控形势和需要，加快建立健全生活物资保障体系，畅通重要生活物资物流通道。在各大中城市科学规划建设一批集仓储、分拣、加工、包装等功能于一体的城郊大仓基地，确保应急状况下及时就近调运生活物资，切实保障消费品流通不断不乱。建立完善重要商品收储和吞吐调节机制，持续做好日常监测和动态调控，落实好粮油肉蛋奶果蔬和大宗商品等保供稳价措施。

　　**（三）**创新消费业态和模式。适应常态化疫情防控需要，促进新型消费，加快线上线下消费有机融合，扩大升级信息消费，培育壮大智慧产品和智慧零售、智慧旅游、智慧广电、智慧养老、智慧家政、数字文化、智能体育、“互联网＋医疗健康”、“互联网＋托育”、“互联网＋家装”等消费新业态。加强商业、文化、旅游、体育、健康、交通等消费跨界融合，积极拓展沉浸式、体验式、互动式消费新场景。有序引导网络直播等规范发展。深入开展国家电子商务示范基地和示范企业创建。深化服务领域东西协作，大力实施消费帮扶，助力中西部地区特别是欠发达地区提升发展能力和消费水平。
　　二、全面创新提质，着力稳住消费基本盘

　　**（四）**积极推进实物消费提质升级。加强农业和制造业商品质量、品牌和标准建设，推动品种培优、品质提升、品牌打造和标准化生产。推进食用农产品承诺达标合格证制度。支持研发生产更多具有自主知识产权、引领科技和消费潮流、应用前景广阔的新产品新设备。畅通制造企业与互联网平台、商贸流通企业产销对接，鼓励发展反向定制（C2M）和个性化设计、柔性化生产。促进老字号创新发展，加强地理标志产品认定、管理和保护，培育更多本土特色品牌。

　　**（五）**加力促进健康养老托育等服务消费。深入发展多层次多样化医疗健康服务，积极发展中医医疗和养生保健等服务，促进医疗健康消费和防护用品消费提质升级。实施智慧助老行动，加快推进适老化改造和智能化产品开发，发展适合老年人消费的旅游、养生、健康咨询、生活照护、慢性病管理等产品和服务，支持开展省际旅居养老合作。加快构建普惠托育服务体系，支持社会力量提供多元化、规范化托育服务，引导市场主体开发更多安全健康的国产婴幼儿用品。

　　**（六）**持续拓展文化和旅游消费。推动中华优秀传统文化传承创新，促进出版、电影、广播电视等高质量发展。大力发展全域旅游，推动红色旅游、休闲度假旅游、工业旅游、旅游演艺等创新发展，促进非遗主题旅游发展。组织实施冰雪旅游发展行动计划。优化完善疫情防控措施，引导公园、景区、体育场馆、文博场馆等改善设施和服务条件、结合实际延长开放时间。鼓励城市群、都市圈等开发跨区域的文化和旅游年票、联票等。深入推进文化和旅游消费试点示范。积极落实带薪休假制度，促进带薪休假与法定节假日、周休日合理分布、均衡配置。

　　**（七）**大力发展绿色消费。增强全民节约意识，反对奢侈浪费和过度消费，形成简约适度、绿色低碳的生活方式和消费模式。推广绿色有机食品、农产品。倡导绿色出行，提高城市公共汽电车、轨道交通出行占比，推动公共服务车辆电动化。推动绿色建筑规模化发展，大力发展装配式建筑，积极推广绿色建材，加快建筑节能改造。支持新能源汽车加快发展。大力发展绿色家装，鼓励消费者更换或新购绿色节能家电、环保家具等家居产品。加快构建废旧物资循环利用体系，推动汽车、家电、家具、电池、电子产品等回收利用，适当放宽废旧物资回收车辆进城、进小区限制。推进商品包装和流通环节包装绿色化、减量化、循环化。开展促进绿色消费试点。广泛开展节约型机关、绿色家庭、绿色社区、绿色出行等创建活动。

　　**（八）**充分挖掘县乡消费潜力。建立完善县域统筹，以县城为中心、乡镇为重点、村为基础的县域商业体系。深入实施“数商兴农”、“快递进村”和“互联网＋”农产品出村进城等工程，进一步盘活供销合作社系统资源，引导社会资源广泛参与，促进渠道和服务下沉。鼓励和引导大型商贸流通企业、电商平台和现代服务企业向农村延伸，推动品牌消费、品质消费进农村。以汽车、家电为重点，引导企业面向农村开展促销，鼓励有条件的地区开展新能源汽车和绿色智能家电下乡，推进充电桩（站）等配套设施建设。提升乡村旅游、休闲农业、文化体验、健康养老、民宿经济、户外运动等服务环境和品质。
　　三、完善支撑体系，不断增强消费发展综合能力

　　**（九）**推进消费平台健康持续发展。加快推进国际消费中心城市培育建设。积极建设一批区域消费中心，改善基础设施和服务环境，提升流通循环效率和消费承载力。支持有条件的地区依托自由贸易试验区等，与国（境）外机构合作建设涉外消费专区。鼓励各地区围绕商业、文化、旅游、体育等主题有序建设一批设施完善、业态丰富、健康绿色的消费集聚区，稳妥有序推进现有步行街设施改造和业态升级，积极发展智慧商圈。推动建设城市一刻钟便民生活圈，优化配置社区生活消费服务综合体。高水平办好“中国品牌日”、全国消费促进月等活动。支持各地区建立促消费常态化机制，培育一批特色活动品牌。持续办好中国国际进口博览会、中国进出口商品交易会、中国国际服务贸易交易会、中国国际消费品博览会。完善市内免税店政策，规划建设一批中国特色市内免税店。

　　**（十）**加快健全消费品流通体系。进一步完善电子商务体系和快递物流配送体系，加强疫情防控措施跨区域相互衔接，畅通物流大通道，加快构建覆盖全球、安全可靠、高效畅通的流通网络。支持智能快件箱（信包箱）、快递服务站进社区，加强末端环节及配套设施建设。加快发展冷链物流，完善国家骨干冷链物流基地设施条件，培育一批专业化生鲜冷链物流龙头企业。大力推广标准化冷藏车，鼓励企业研发应用适合果蔬等农产品的单元化包装，推动实现全程“不倒托”、“不倒箱”。健全进口冷链食品检验检疫制度，加快区块链技术在冷链物流智慧监测追溯系统建设中的应用，推动全链条闭环追溯管理，提高食品药品流通效率和安全水平。针对进口物品等可能引发的输入性疫情，严格排查入境、仓储、加工、运输、销售等环节，建立健全进口冻品集中监管制度，筑牢疫情外防输入防线。

　　**（十一）**增加就业收入提高消费能力。鼓励创业带动就业，支持各类劳动力市场、人才市场、零工市场建设，支持个体经营发展，增加非全日制就业机会，规范发展新就业形态，健全灵活就业劳动用工和社会保障政策。实施提升就业服务质量工程，加强困难人员就业帮扶，完善职业教育体系，开展大规模、多层次职业技能培训，加大普惠性人力资本投入力度。解决好高校毕业生等青年就业问题。健全工资决定、合理增长和支付保障机制，稳步提高劳动者工资性收入特别是城市工薪阶层、农民工收入水平，健全最低工资标准调整机制。接续推进乡村富民产业发展，落实和完善对农民直接补贴政策，拓宽乡村特别是脱贫地区农民稳定就业和持续增收渠道。

　　**（十二）**合理增加公共消费。健全常住地提供基本公共服务制度，合理确定保障标准。紧扣人民群众“急难愁盼”，多元扩大普惠性非基本公共服务供给。提高教育、医疗、养老、育幼等公共服务支出效率。完善长租房政策，扩大保障性租赁住房供给。支持缴存人提取住房公积金用于租赁住房，继续支持城镇老旧小区居民提取住房公积金用于加装电梯等自住住房改造。健全基本生活救助制度和专项救助制度，积极发展服务类社会救助，形成“物质＋服务”的多样化综合救助方式。落实好社会救助和保障标准与物价上涨挂钩联动机制。
　　四、持续深化改革，全力营造安全放心诚信消费环境

　　**（十三）**破除限制消费障碍壁垒。有序破除一些重点服务消费领域的体制机制障碍和隐性壁垒，促进不同地区和行业标准、规则、政策协调统一，简化优化相关证照或证明办理流程手续。稳定增加汽车等大宗消费，各地区不得新增汽车限购措施，已实施限购的地区逐步增加汽车增量指标数量、放宽购车人员资格限制，鼓励除个别超大城市外的限购地区实施城区、郊区指标差异化政策，更多通过法律、经济和科技手段调节汽车使用，因地制宜逐步取消汽车限购，推动汽车等消费品由购买管理向使用管理转变。建立健全汽车改装行业管理机制，加快发展汽车后市场。全面取消二手车限迁政策，落实小型非营运二手车交易登记跨省通办措施。对皮卡车进城实施精细化管理，研究进一步放宽皮卡车进城限制。

　　**（十四）**健全消费标准体系。健全消费品质量标准体系，大力推动产品质量分级。完善节能和绿色制造标准体系、绿色产品认证标识体系以及平台经济、跨境电商、旅游度假、餐饮、养老、冷链物流等领域服务标准。推进第五代移动通信（5G）、物联网、云计算、人工智能、区块链、大数据等领域标准研制，加快超高清视频、互动视频、沉浸式视频、云游戏、虚拟现实、增强现实、可穿戴等技术标准预研，加强与相关应用标准的衔接配套。

　　**（十五）**加强消费领域执法监管。深入实施公平竞争政策，强化反垄断和反不正当竞争执法，加快建立健全全方位、多层次、立体化监管体系，防止资本无序扩张。加大对虚假宣传、仿冒混淆、制假售假、缺斤短两等违法行为的监管和处罚力度。全面加强跨地区、跨部门、全流程协同监管，压实生产、流通、销售等各环节监管责任。加快消费信用体系建设，推进信用分级分类监管，组织开展诚信计量示范活动，依法依规实施失信惩戒。加强价格监管，严厉打击低价倾销、价格欺诈等违法行为，严格规范平台经营者自主定价。继续加强消费品质量安全监管，开展消费品质量合格率统计调查，加大缺陷产品召回监管力度。加强重点服务领域质量监测评价。

　　**（十六）**全面加强消费者权益保护。大力开展放心消费创建活动。完善平台经济消费者权益保护规则。持续优化完善全国12315平台，充分发挥地方12345政务服务便民热线作用，进一步畅通消费者投诉举报渠道。建立完善消费投诉信息公示制度。进一步优化消费争议多元化解机制，不断提升在线消费纠纷解决机制效能。完善公益诉讼制度，探索建立消费者集体诉讼制度，全面推行消费争议先行赔付。广泛引导线下实体店积极开展无理由退货承诺。
　　五、强化保障措施，进一步夯实消费高质量发展基础

　　**（十七）**加强财税支持。统筹利用现有财政资金渠道，支持消费相关基础设施和服务保障能力建设，符合条件的项目可纳入地方政府专项债券支持范围，更好以投资带消费。完善政府绿色采购标准，加大绿色低碳产品采购力度。鼓励有条件的地区对绿色智能家电、绿色建材、节能产品等消费予以适当补贴或贷款贴息。研究进一步降低与人民生活密切相关、需求旺盛的优质消费品进口关税。

　　**（十八）**优化金融服务。引导银行机构积极发展普惠金融，探索将真实银行流水、第三方平台收款数据、预订派单数据等作为无抵押贷款授信审批参考依据，提高信用状况良好的中小微企业和消费者贷款可得性。推动商业银行、汽车金融公司、消费金融公司等提升金融服务能力。强化县域银行机构服务“三农”的激励约束机制，丰富农村消费信贷产品和服务，加大对农村商贸流通和居民消费的金融支持力度。引导金融机构在风险可控和商业可持续前提下丰富大宗消费金融产品。鼓励保险公司针对消费领域提供保险服务。规范互联网平台等涉及中小微企业、个体工商户金融服务的收费行为。

　　**（十九）**强化用地用房保障。加大土地、房屋节约集约和复合利用力度，鼓励经营困难的百货店、老旧厂区等改造为新型消费载体。鼓励通过先租后让、租让结合等方式为快递物流企业提供土地。适应乡村旅游、民宿、户外运动营地及相关基础设施建设小规模用地需要，积极探索适宜供地方式，鼓励相关设施融合集聚建设。优化国有物业资源出租管理，适当延长租赁期限，更好满足超市、便利店等消费场所用地用房需求。支持利用社区存量房产、闲置房屋等建设便民网点。允许有条件的社区利用周边空闲土地或划定的特定空间有序发展旧货市场。

　　**（二十）**压实各方责任。国家发展改革委、商务部等有关部门要充分发挥完善促进消费体制机制部际联席会议制度作用，强化协同联动，加强督办落实。国家统计局要完善服务消费统计监测，建立健全网络消费等消费新业态新模式统计体系。各地区要加强组织领导，完善配套方案，切实推动本意见提出的各项任务措施落地见效。

国务院办公厅
　　2022年4月20日

© 北大法宝：（[www.pkulaw.com](https://www.pkulaw.com/)) 专业提供法律信息、法学知识和法律软件领域各类解决方案。北大法宝为您提供丰富的参考资料，正式引用法规条文时请与标准文本核对。
欢迎查看所有[产品和服务](https://www.pkulaw.com/helps/2.html)。法宝快讯：[如何快速找到您需要的检索结果？](https://www.pkulaw.com/helps/70.html)[法宝](https://www.pkulaw.com/helps/69.html)V6有何新特色？

扫描二维码阅读原文

**原文链接：**<https://www.pkulaw.com/chl/1b66c4a8e34fccfebdfb.html>
